# Emergence of spin–orbit fields in magnetotransport of quasi-two-dimensional iron on gallium arsenide

**DOI:** 10.1038/ncomms8374

**Published:** 2015-06-08

**Authors:** T. Hupfauer, A. Matos-Abiague, M. Gmitra, F. Schiller, J. Loher, D. Bougeard, C. H. Back, J. Fabian, D. Weiss

**Affiliations:** 1 Institute of Experimental and Applied Physics, University of Regensburg, 93040 Regensburg, Germany; 2 Institute of Theoretical Physics, University of Regensburg, 93040 Regensburg, Germany

## Abstract

The desire for higher information capacities drives the components of electronic devices to ever smaller dimensions so that device properties are determined increasingly more by interfaces than by the bulk structure of the constituent materials. Spintronic devices, especially, benefit from the presence of interfaces—the reduced structural symmetry creates emergent spin–orbit fields that offer novel possibilities to control device functionalities. But where does the bulk end, and the interface begin? Here we trace the interface-to-bulk transition, and follow the emergence of the interfacial spin–orbit fields, in the conducting states of a few monolayers of iron on top of gallium arsenide. We observe the transition from the interface- to bulk-induced lateral crystalline magnetoanisotropy, each having a characteristic symmetry pattern, as the epitaxially grown iron channel increases from four to eight monolayers. Setting the upper limit on the width of the interface-imprinted conducting channel is an important step towards an active control of interfacial spin–orbit fields.

## Abstract (web summary)

Broken symmetry at material interfaces allows for novel spintronic functionality via emergent spin–orbit effects. Here, Hupfauer *et al*. follow the interface-to-bulk transition of ultra-thin epitaxial iron films on gallium arsenide via anisotropic magnetoresistance measurements and first-principle calculations.

## Graphical abstract

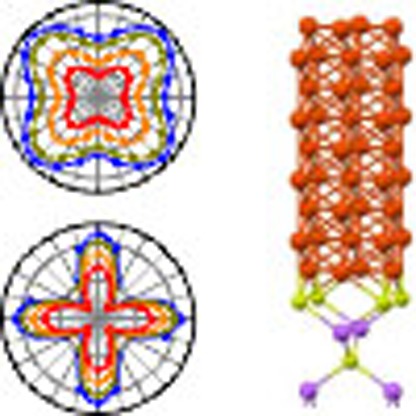

In recent years, spintronics research^1^ has been fuelled by a new class of experiments related to the combined interaction of the spin and orbital degree of freedom^2^,^3^,^4^,^5^,^6^,^7^,^8^,^9^,^10^. The reduced structural symmetry at surfaces and interfaces gives rise to emergent spin–orbit fields (SOF)^11^. Interfacial SOF are important for phenomena such as tunnelling anisotropic magnetoresistance (AMR)^12^,^13^, current driven torques at interfaces^7^,^8^,^14^,^15^,^16^, formation of two-dimensional magnetic skyrmion lattices^17^ or the realization of topological superconductors^18^.

Owing to the lack of space inversion symmetry at the interface, the electronic structure changes qualitatively—the electronic states are spin split. Unlike a magnetic field, the SOF depend on the electron momentum **k**, ensuring the time reversal invariance of spin–orbit coupling. Two most common SOFs are (two-dimensional) Dresselhaus^19^,^20^, **w**_D_=*β*(*k*_*y*_, *k*_*x*_), and the Bychkov–Rashba^21^,^20^, **w**_BR_=*α*(−*k*_*y*_, *k*_*x*_) fields, originally introduced to describe the spin–orbit coupling effects in two-dimensional electron gases formed in zinc-blende semiconductors grown along [001]. Real parameters *α* and *β* characterize the strength of the two fields. Combined, the two fields describe a more general C_2v_ SOF appearing in solids with the C_2v_ point symmetry^20^.

Fe on GaAs, grown along [001] has C_2v_ symmetry, induced by the GaAs surface. The same SOFs are also the basis for the evolution of the magnetocrystalline anisotropy in ultra-thin films of Fe/GaAs(001) (refs 22, 23). Electrons in Fe should then experience the Dresselhaus and Bychkov–Rashba fields as well. For structures with thick Fe layers (for which magnetotransport was previously studied^24^,^25^), the interface-induced SOF would be hardly noticed, but for ultra-thin films of Fe, consisting of a few atomic monolayers, the interfacial symmetry could even dominate. This is precisely what we observe in our samples of epitaxially grown Fe on GaAs. As the thickness of Fe layers decreases, the symmetry of the AMR, which is a signature of spin–orbit coupling, evolves from four-fold—Fe bulk like—to two-fold C_2v_—interface like. We observe reorientation of the main symmetry axes when decreasing the Fe thickness, as well as when increasing temperature. The simple d.c.-transport and measured AMR, presented below, give direct evidence of the presence of the interfacial SOFs. A phenomenological theory, motivated and supported by first-principles calculations, yields perfect fits to the experimental data, allowing us to quantify both the interface and bulk contributions to AMR.

## Results

### Experimental set-up

**Figure 1:**
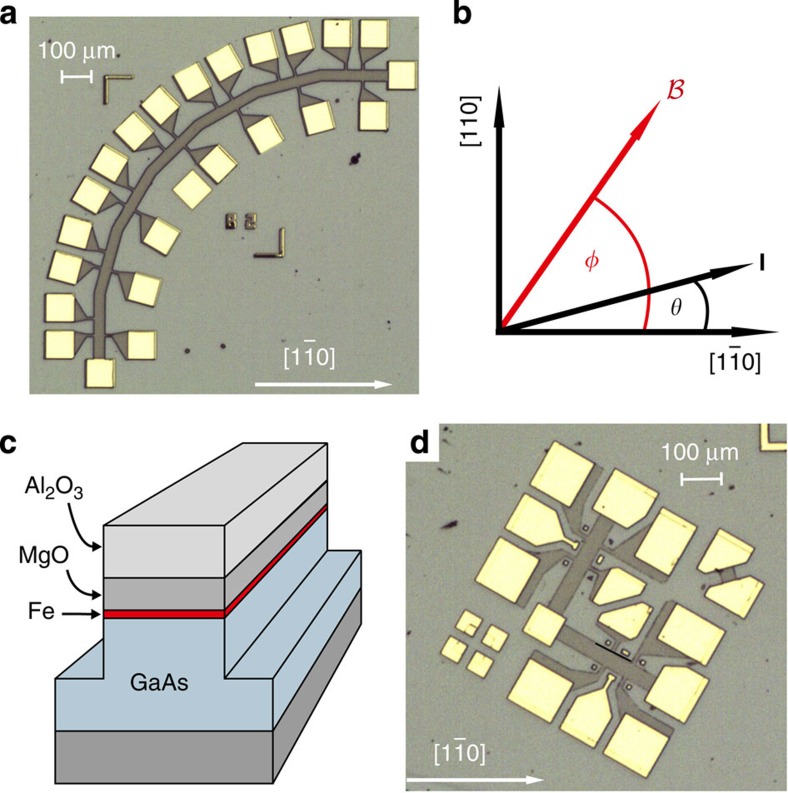
Design of the Fe/GaAs devices. (**a**) Optical micrograph of the quadrant type sample after etching and contact metallization. The mesa, defining the transport channel (current direction), is aligned along seven different crystallographic directions allowing to measure simultaneously the corresponding voltage drops. The 
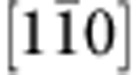
 direction is given by the white arrow and the white bar defines the length scale. (**b**) The angles *θ* and *φ* define, respectively, the direction of the current and the magnetic field with respect to the hard magnetic axis (
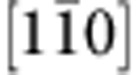
). (**c**) Sketch of the mesa and the layer sequence. (**d**) Optical micrograph of an L-shaped sample after etching and contact metallization. To cover all crystallographic directions in 15°-steps 6 of these structures with different *θ* were fabricated.

We studied four, six and eight monolayers of Fe epitaxially grown by molecular beam epitaxy (MBE) onto a 100-nm thick layer of undoped GaAs. The mesa structures fabricated from these Fe/GaAs heterostructures, shown in Fig. 1, were defined employing optical lithography and ion beam etching. We used two geometries—a quadrant type sample with the transport channel aligned along seven different crystallographic directions shown in Fig. 1a and an L-shaped one with the transport channels aligned in two perpendicular directions, displayed in Fig. 1d.

For both sample types, the Hall bar width is 40 μm and the separation of the potential probes is 50 μm. For each Fe film thickness, we fabricated and investigated several devices that all show basically the same result.

The AMR measurements were carried out in a variable temperature insert of a ^4^He cryostat at temperatures ranging from *T*=1.8 to 150 K. The samples were mounted in a sample holder, rotatable by 360° in an in-plane constant external magnetic field produced by superconducting magnet coils. The angles *θ* and *φ* in the plane of the film (see Fig. 1b) refer, respectively, to the directions of the current path and the magnetic field with respect to the 
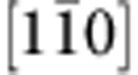
-direction of the GaAs crystal. To vary the current direction *θ* by 360°, we used, besides the quadrant sample, shown in Fig. 1a, the L-shaped ones, shown in Fig. 1d. By reversing the current direction, we obtain with two quadrant samples a rotation of **j** in steps of 15°; using the L-shaped geometry instead, six different samples are needed to cover the full *θ* dependence.

### First-principles calculations

**Figure 2:**
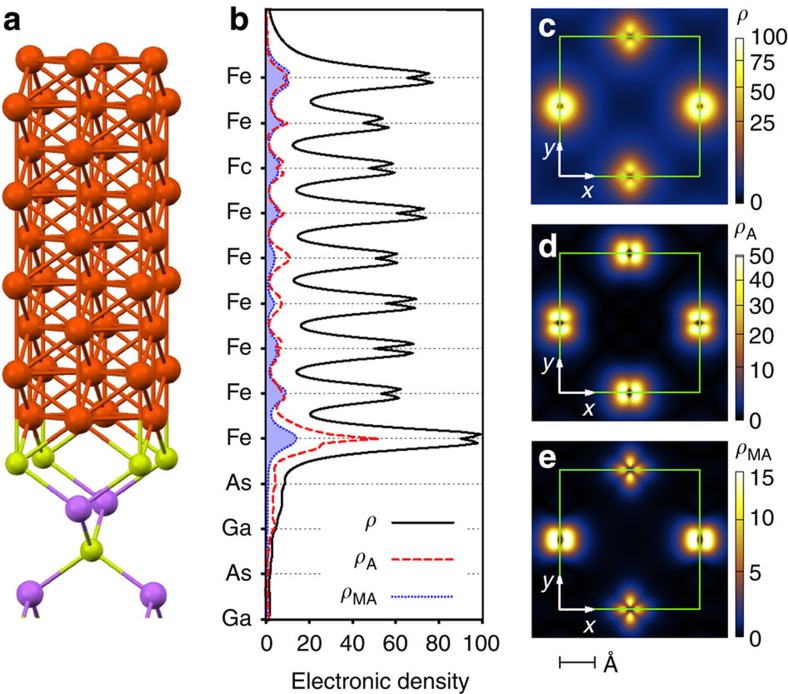
Anisotropy of the Fe/GaAs interface from first principles. (**a**) Atomic structure of the interface with nine Fe layers. (**b**) Calculated electronic density *ρ*(*z*), anisotropic electronic density *ρ*(*z*)_A_ and magnetoanisotropic electronic density *ρ*(*z*)_MA_ for the 10-meV window at the Fermi level. The plots are rescaled by the same factor to assign the value of 100 to the maximum of *ρ*(*z*). (**c**), *ρ*(*x*,*y*) (which sets the scale in the same way), (**d**) *ρ*(*x*,*y*)_A_ and (**e**) *ρ*(*x*,*y*)_MA_ in the plane of the interfacial Fe atom. The largest densities occur in the vicinity of the four Fe atoms within a unit cell (green). Anisotropic charge density *ρ*_A_ is calculated for magnetization along [110].

To gain insight into what anisotropy of the electronic structure can be expected from the Fe/GaAs interface, we have performed first-principles calculations (see also Supplementary Note 1, Supplementary Figs 1 and 2) of various Fe/GaAs slabs. Figure 2 shows the results for a slab with nine Fe monolayers. The electronic density *ρ*(*z*), averaged in the (*x*, *y*) plane, is shown in parallel to the atomic structure. The orientation of the axes is as follows: 
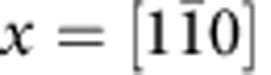
, *y*=[110] and *z*=[001], as in the experiment Fig. 1. To quantify the C_2v_ anisotropy due to the interface, we introduce two measures—the anisotropy of the charge density,


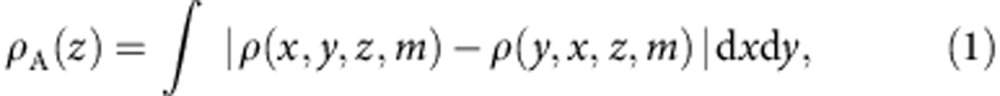


and the magnetoanisotropy of the charge density


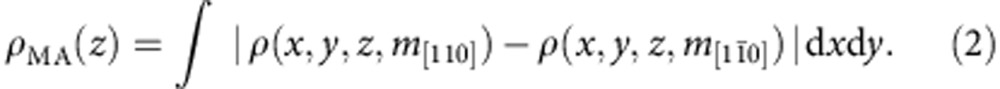


Here *m* denotes the orientation of the magnetization. The formula for *ρ*_A_ is motivated by the fact that interchanging the principal symmetry axes *x* and *y* is not a symmetry operation of C_2v_, while it is under the four-fold C_4v_. The magnetoanisotropy *ρ*_MA_ is determined by the differences in the charge densities when the orientation of magnetization changes from [110] to 
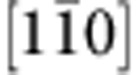
. For a four-fold symmetry, both *ρ*_A_ and *ρ*_MA_ vanish. The anisotropy *ρ*_A_ is sensitive mainly to the orbital effects of the interface, while magnetoanisotropy *ρ*_MA_ is due to the interfacial spin–orbit coupling. Both *ρ*_A_(*z*) and *ρ*_MA_(*z*) are shown in Fig. 2b, relative to the charge density *ρ*(*z*), for the states in a 10-meV window at the Fermi level. The anisotropies are about 10%. The largest anisotropy is at the interfacial Fe monolayer. In fact, it is the penetration of the Fe Fermi-level Bloch states into the interface region that imprints the interfacial C_2v_ symmetry onto the lateral Fe transport. This penetration is nicely seen in Fig. 2b. As the Fe thickness decreases, the two-fold anisotropies steadily increase (see Supplementary Note 1, Supplementary Table 1). In Fig. 2c–e, we plot the cross-sectional charge density *ρ*(*x*, *y*), and the corresponding anisotropies *ρ*(*x*, *y*)_A_=|*ρ*(*x*, *y*, *m*)−*ρ*(*y*, *x*, *m*)| for *m*=[110] and *ρ*(*x*, *y*)_MA_=|*ρ*(*x*, *y*, *m*=*x*)−*ρ*(*x*, *y*, *m*=*y*)| for the plane of the interfacial Fe, respectively, resolving the d-character of the atomic orbitals of Fe. The existence of SOFs in Fe/GaAs was demonstrated from first principles in ref. 26, ramifications for magnetooptics were worked out in ref. 27.

### Anisotropic resistance and magnetoresistance

**Figure 3:**
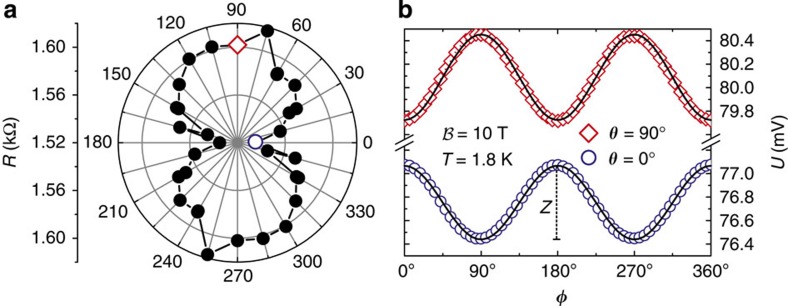
Resistance and magnetoresistance anisotropy. (**a**) Average resistance for different current directions *θ* of a sample with 6 ML Fe. The resistance anisotropy is ∼4.3%. (**b**) Measurement of the longitudinal voltage drop for a constant current of 50 μA as a function of the external magnetic field (magnetization) direction *φ*, rotated in the interfacial plane. Data were taken at 1.8 K employing the L-shaped mesas. The two highlighted data points in the polar plot correspond to the two current directions shown in the graph to the right, featuring a difference in the average resistance. Importantly, the AMR effect shows a small but systematic difference in amplitude *Z*=*U*_max_−*U*_min_ for the two current directions along 
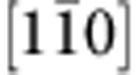
 (*θ*=0°) and [110] (*θ*=90°). The solid lines in **b** correspond to a theoretical fit to the experimental data (symbols).

Typical experimental results are displayed in Fig. 3. The resistance averaged over all magnetization directions depends on the crystallographic direction and is always smallest for **j** flowing along the 
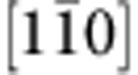
 direction (*θ*=0°). This is shown for devices with six-monolayer Fe in Fig. 3a, for which the data were taken from eight different L-shaped samples. The four-monolayer sample shows a similar resistance anisotropy, while the change in resistance of the eight-monolayer device is comparable to the noise level. This anisotropy of the average resistance is expected and stems, as is pointed out below, from the anisotropy of the charge density *ρ*_A_ at the Fe/GaAs interface. For the six-monolayer sample, displayed in Fig. 3a, the resistance anisotropy is about 4.3%, to be compared with the calculated anisotropy of about 25% for an ideal Fe/GaAs interface (see Supplementary Table 1). We now turn to the central experimental observation being the *θ* and *φ* dependence of the AMR, an effect directly connected to the spin–orbit coupling. Figure 3b displays the AMR signal, that is, the longitudinal voltage drop as a function of the in-plane magnetization direction *φ* for constant current *I*=50 μA. In experiment, the magnetization direction was rotated by a strong external magnetic field of 10 T strength. The voltages *U*(*φ*) shown correspond to *θ*=0° and 90°, that is, for a current flowing along the 
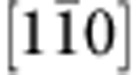
 and [110] direction, respectively, and are highlighted in Fig. 3a. The signal measured for both directions exhibits a cos^2^*φ* dependence, typical for AMR. Most importantly the AMR is different for the two current directions: its amplitude *Z* is ∼0.736 mV for *θ*=90° and ∼0.628 mV for *θ*=0°. From that we conclude that the AMR depends on the crystallographic direction of the current flow (*θ*) allowing us below to map out the symmetry of the interfacial SOFs.

### Crystalline AMR

To quantify the AMR amplitude as a function of θ we introduce the crystalline AMR (CAMR) coefficient,


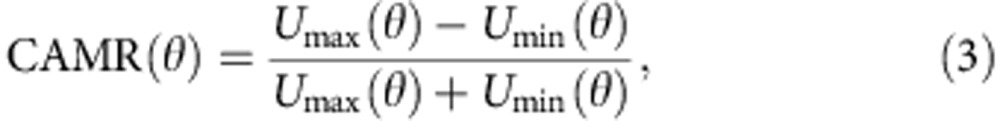


where *U*_max_ (*U*_min_) refers to the maximum (minimum) value of the voltage drop obtained when *φ*=*θ* (*φ*=*θ*+90°). To experimentally extract information about the interfacial SOFs, measurements like the one shown in Fig. 3b were systematically carried out varying the number of Fe monolayers, temperature and direction of current flow *θ*. We would like to stress that the CAMR effect discussed here originates from the interfacial SOF and not, as, for example, in (Ga,Mn)As layers^28^,^29^,^30^,^31^, from bulk spin–orbit interaction.

**Figure 4:**
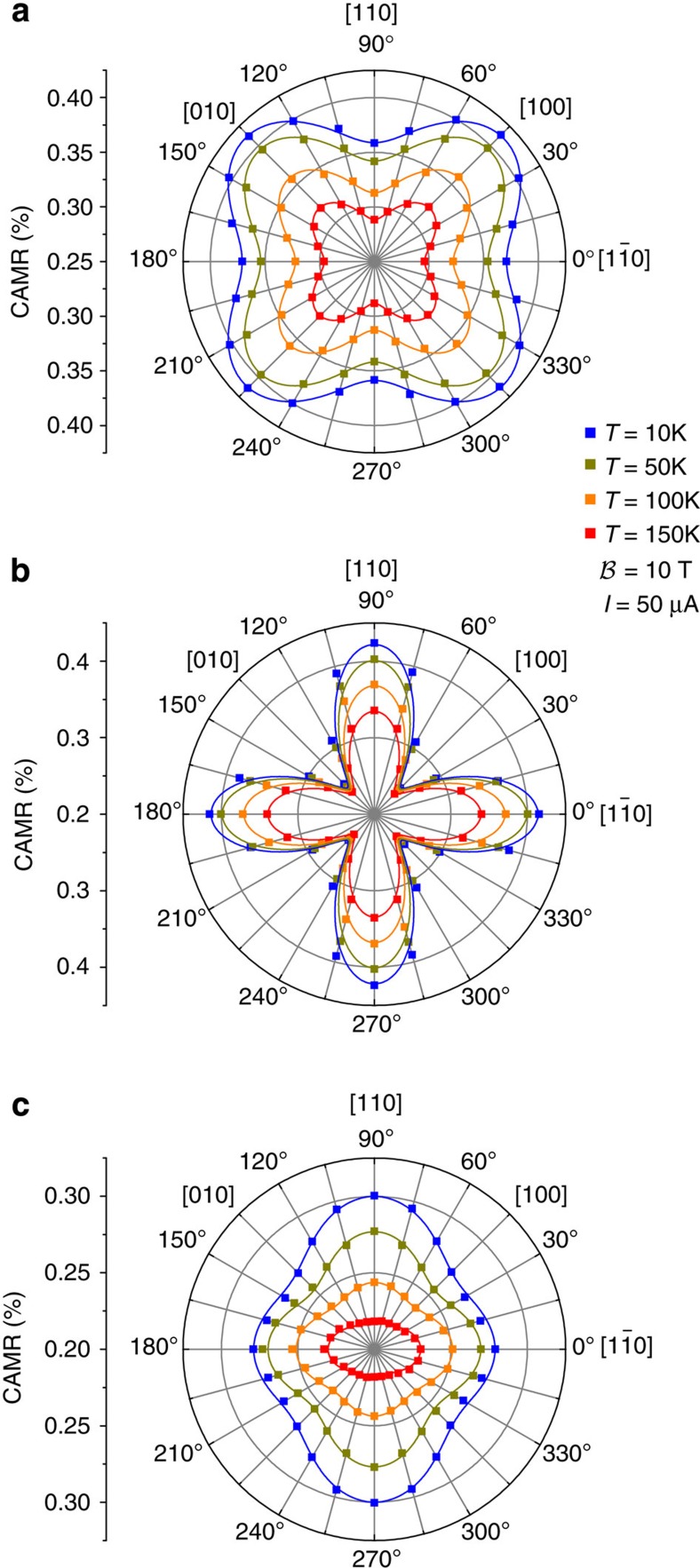
Crystalline AMR. The CAMR is shown as a function of the direction of the current flow (*θ*) at different temperatures. (**a**–**c**) correspond to samples with eight, six and four monolayers of Fe, respectively. The symbols are experimental data. The solid lines are fits using our phenomenological model. The difference between the maxima of the CAMR along 
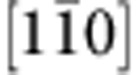
 and [110] accounts for the presence of the interface SOF, which lowers the symmetry from four-fold to two-fold.

The measured CAMR as a function of the current direction, *θ* is shown in Fig. 4 for different Fe thicknesses and temperatures. The data were taken from two quadrant type samples allowing to change the angle *θ* in 15° steps. In all the cases, the CAMR exhibits a two-fold symmetry with maxima (minima for the case of eight monolayers) for currents flowing along the 
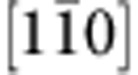
 and [110] directions and minima (maxima for the case of eight monolayers) for currents along the [100] and [010] directions. CAMR ranges from 0.2 to 0.4%, decreasing with increasing temperature. The two-fold C_2v_ symmetry component is dominant for the thinnest, four-monolayer, sample. The six-monolayer sample has a strong four-fold component, with the symmetry axes along the 
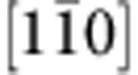
 and [110] directions, the principal axes of the interfacial C_2v_. Only in the eight-monolayer sample we observe the dominant four-fold symmetry from bulk Fe, with principal axes [100] and [010].

### Phenomenological model

The evolution of the symmetry of AMR, from interface to bulk like, with increasing the Fe layer thickness, can be quantitatively described by a phenomenological model which introduces the interfacial C_2v_ SOFs as a perturbation to a bcc (Fe) system. An analogous model was used to describe tunnelling AMR in Fe/GaAs tunnelling devices^12^,^32^. In short, our system is characterized by two vectors—magnetization **m** and SOF **w**(**k**). The momentum-resolved conductivity tensor *g* can be expanded in the direction cosines of **m**, as well as in the components of the SOF, up to the second powers. The expansion parameters are restricted by the cubic O_h_ symmetry of the unperturbed system; the conductivity tensor also obeys the Onsager relation *g*(**m**)=*g*^T^(−**m**). Averaging over the Fermi momenta, considering that the SOFs are odd in momenta, **w**(**k**)=−**w**(−**k**) (so that averages of terms linear in **w** vanish), then gives the conductivity tensor for the lateral transport in Fe, influenced by the SOFs due to the interface. Leaving the details for Supplementary Note 2, we give the result for the longitudinal resistance:


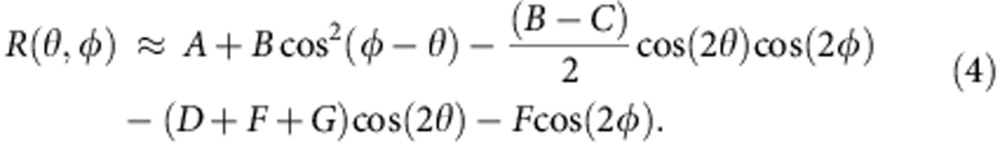


The constants *A*, *B* and *C*, are, in general, different from zero, independent of the presence or not of the interface SOC field. Therefore, they do not account for the interface-induced anisotropy. In particular, *A* is the isotropic resistance, *B* is the conventional AMR that depends only on the relative angle between current and magnetization direction; *C* depends on both the bulk Fe spin–orbit coupling as well as on the magnitude (not direction) of interfacial SOFs, so it does not carry information on the C_2v_ symmetry; *D* has two contributions, one exclusively determined by the spin–orbit coupling and one combining SOF field and magnetism. Thus, in dependence of the symmetry of the SOF, *D* can be finite even in the absence of magnetism (it can therefore account for anisotropic transport in two-dimensional electron gases in zinc-blende semiconductors^33^,^34^). The parameter *F* contains information of magnetism and SOF. It combines the crystalline anisotropy produced by SOF with the magnetic properties of the system and, as shown below, characterizes the interface-induced CAMR. Finally, *G* has a purely orbital nature and describes the intrinsic anisotropy of the interfacial structure.

Using the symmetry properties of the Dresselhaus and Bychkov–Rashba SOFs, one finds *D* and *F* proportional to the product *αβ* (refs 20, 35). Therefore, for a C_2v_ symmetric SOF (that is, *αβ*≠0), the parameters *D* and *F* are, in general, finite. In contrast, if the symmetry of the SOC is C_∞v_ (*β*=0), D_2d_ (*α*=0) or C_4v_ (terms of higher order in momentum reduce the symmetry of the Bychkov–Rashba SOF to C_4v_), both *D* and *F* vanish.

## Discussion

The experimentally found behaviour of the CAMR can be understood in terms of our phenomenological model. Using equations (3) and (4), and taking into account that the anisotropic corrections are small, we obtain


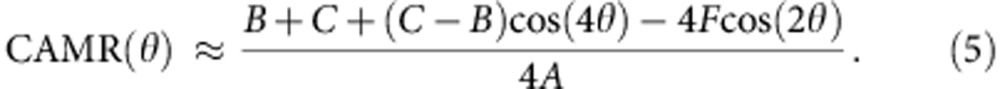


This expression fits perfectly the experimental data in Fig. 4. Equation (5) provides insight about the microscopic origin of the CAMR. The amplitude of the four-fold symmetric contribution [∝cos(4*θ*)] is determined by the phenomenological parameters *B* and *C* which, as discussed above, are finite even in the absence of interfacial SOFs. The four-fold term then accounts for the cubic symmetry of the underlying Fe system in the absence of the interface and is responsible for the appearance of the four lobes in the polar plots of the CAMR (see Fig. 4). On the other hand, the amplitude of the two-fold symmetric contribution [∝cos(2*θ*)] is given by the parameter *F* only which, as discussed above, accounts for the C_2v_ SOFs of the Fe/GaAs interface and increases with the wave function penetration into the interface region. This contribution is responsible for the observed difference between the maxima of the CAMR along 
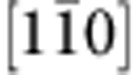
 and [110]. In fact, it follows from equations (5) that CAMR(90°)−CAMR(0°) ∝*F*.

Our model also explains the reorientation of the four-fold symmetry axes when increasing the Fe thickness from six to eight monolayers (see Fig. 4). Which axes become principal depends on the sign of *C*−*B* in equation (5). Both *B* and *C* depend on the bulk Fe properties, as well as on the amplitude (but not direction) of the interfacial SOFs. The term corresponding to the bulk Fe properties dominates in thicker samples (eight monolayers), while the interfacial one in thinner samples (six monolayers). The two contributions compete, giving the sign change of *C*−*B* when changing the layer thickness, causing reorientation of the CAMR symmetry axes, in agreement with the experimental observation. Further details can be found in the Supplementary Note 3, and the extracted values of the phenomenological parameters and their temperature dependence, are presented in Supplementary Figs 3–5.

In conclusion, we have given experimental evidence, from a simple d.c. transport measurement, of interfacial SOFs in ultra-thin Fe on GaAs. We have also given a general phenomenological model that nicely fits all the experimental data, and qualitatively accounts for the observed evolution of the CAMR from interface dominated two-fold, via an intermediate one with the symmetry axes still given by the interfacial symmetry, to the four-fold bulk dominated symmetry, as the thickness of the Fe layer increases from four, through six, to eight monolayers. Our findings open the field of lateral anisotropic magnetotransport in ferromagnetic nanochannels on zinc-blende semiconductors, which has potential technological applications in memory-sensing and memory-storage devices.

## Methods

### Experimental methods

The thin Fe films of varying thickness were grown by MBE at a temperature of ∼75 °C side by side on the same surface by controlling the monolayer growth using a shiftable shutter. The GaAs layer had been grown before in a separate chamber onto a (001)-oriented semi-insulating GaAs substrate using an excess of As, which ensures an As-terminated surface, showing a (2 × 4) surface reconstruction. The As termination ensures that Ga diffusion into the Fe film is kept to a minimum and justifies the assumed As termination in the *ab initio* calculations. For Fe deposition, the GaAs wafer was transferred into the attached metal MBE chamber. Flat and epitaxial growth of Fe was confirmed by monitoring reflection high-energy electron diffraction oscillations during Fe film growth. To protect the Fe layer from oxidation, it was covered with an ∼10-nm-thick layer of thermally evaporated MgO and 15 nm Al_2_O_3_, grown by atomic layer deposition. The mesa structures fabricated from these Fe/GaAs heterostructures were defined employing optical lithography and ion beam etching. The contact pads, also defined by optical lithography and consisting of 15 nm Ti and 130 nm Au, were evaporated on top of the Al_2_O_3_ and contact to the Fe layer was made by ultrasonic bonding with sufficiently high power to penetrate the insulating layers. This results in ohmic contacts with negligible contact resistance of <1 kΩ, confirmed by comparing two- and four-wire *I*–*V* characteristics at cryogenic and room temperature. All these steps, except the final Al_2_O_3_-deposition, were performed without breaking the vacuum.

### Theoretical methods

The first-principles calculations reported in the manuscript were performed using the full potential linearized augmented planewave method as implemented in Wien2k code^36^. The method is an all-electron method within density functional theory^37^. We used a generalized gradient approximation^38^ for the exchange-correlation functional. We considered muffin-tin radii 2.3, 1.89 and 2.3 Bohr radii for Ga, As and Fe, respectively. We used mixed linearized augmented planewave and augmented plane wave+local orbital basis set with plane wave energy cutoff of about 24 Ry. For irreducible part of the first Brillouin zone sampling, we considered 364 *k*-points. It turned out that the used parameters yield sufficient accuracy in determining the in-plane magnetocrystalline anisotropy 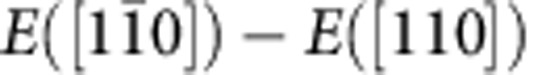 of 0.0065, 0.0057 and 0.0033, mRy for three, six and nine atomic layers of epitaxial Fe on (001) GaAs, respectively. Spin–orbit coupling of the valence electrons has been treated scalar relativistically within the second variational method.

The small lattice mismatch between twice the lattice constant of Fe (2.87 Å) and GaAs (5.65 Å) allows for a smooth epitaxial growth of Fe on a GaAs (001) surface. We took for the diagonal lattice spacing 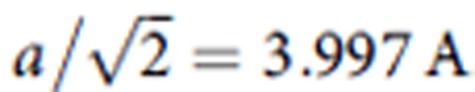. To study the electronic structure of the GaAs/Fe interface, we consider thin ideal Fe/GaAs slabs separated by a vacuum of 13 Å in growth direction (001) to exclude interaction between periodic images. The semiconducting substrate contains 13 atomic layers of GaAs (three unit cells of bulk GaAs) with As-terminated surface and interface. Dangling bonds on surface As atoms have been passivated by two fictitious hydrogen atoms with fractionally reduced proton charge and corresponding fractional electronic charge to 0.75*e*, where *e* is the elementary charge. In this way, irrelevant surface states are removed from the bandgap region due to the formation of the covalent bonds^39^,^40^. The As–H bond length of 1.55 Å and angle 112.9° between Ga, As and H have been obtained from the surface relaxation. For the epitaxially grown Fe on (001) GaAs surface, we consider flat 1 × 1 interface.

## Additional Information

**How to cite this article**: Hupfauer, T. *et al*. Emergence of spin–orbit fields in magnetotransport of quasi-two-dimensional iron on gallium arsenide. *Nat. Commun.* 6:7374 doi: 10.1038/ncomms8374 (2015).

## Supplementary Material

Supplementary Information Supplementary Figures 1-5, Supplementary Table 1, Supplementary Notes 1-3 and Supplementary References
