# Supplementary material for: Emergence of spin–orbit fields in magnetotransport of quasi-two-dimensional iron on gallium arsenide
Source: Nat Commun. 2015 Jun 8;6:7374. doi: 10.1038/ncomms8374 (PMC4468871; doi:10.1038/ncomms8374)
Supplement: Supplementary Information — Supplementary Figures 1-5, Supplementary Table 1, Supplementary Notes 1-3 and Supplementary References [file ncomms8374-s1.pdf]

Supplementary Information

Supplementary Figures

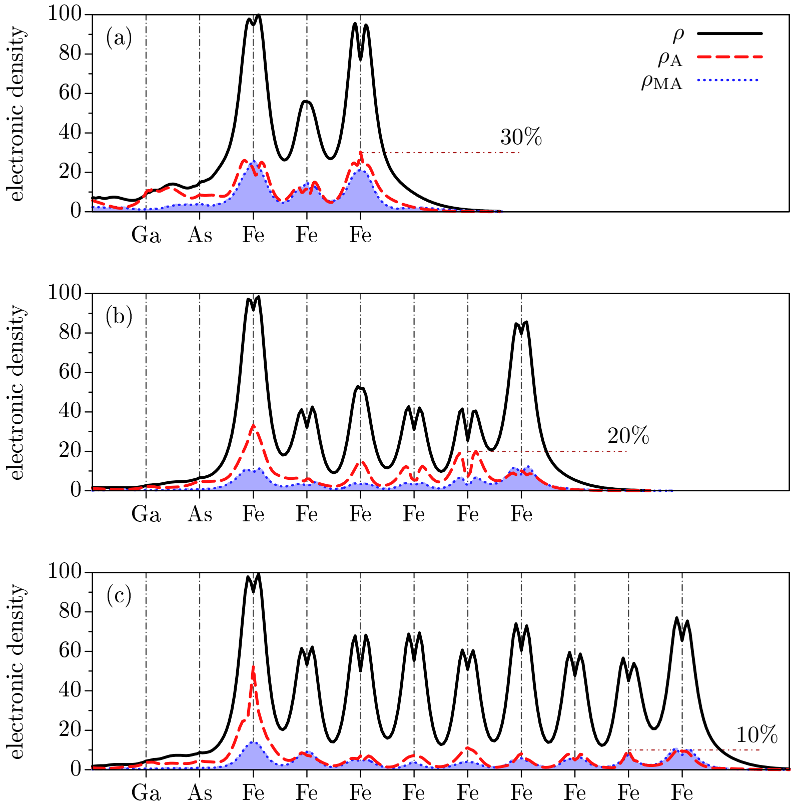

Supplementary Figure 1: **Anisotropy and magnetoanisotropy of charge density**. Calculated plane averaged electronic density $\rho(z)$ in the energy window of ± 5 meV around the Fermi level, the anisotropy of the density ρ_A, and the magnetic anisotropy ρ_{MA} for (a) three, (b) six, and (c) nine atomic layers of Fe. The plots are rescaled by the same factor to assign the value of 100 to the maximum of $\rho(z)$.

.

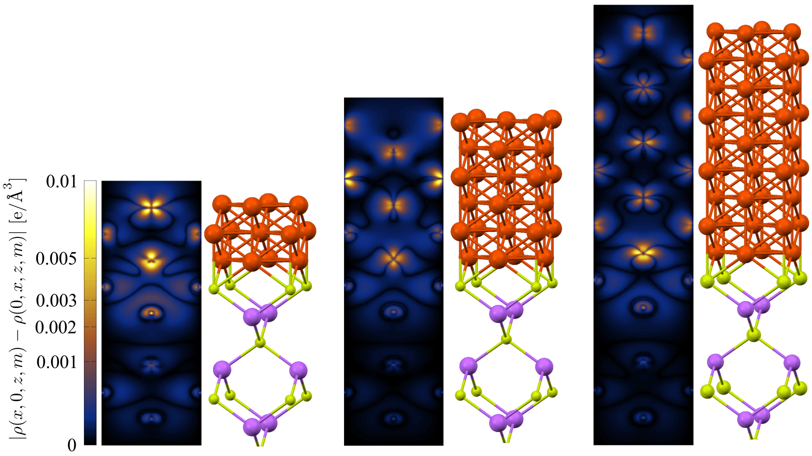

Supplementary Figure 2: **Anisotropic density and atomic structure**. Anisotropy of the electronic density calculated in energy window of ± 5 meV around Fermi level at xz plane cross section for studied slabs of three, six, and nine atomic layers of the epitaxial Fe. Corresponding atomic structures are also shown. $m = [1\bar{1}0]$.

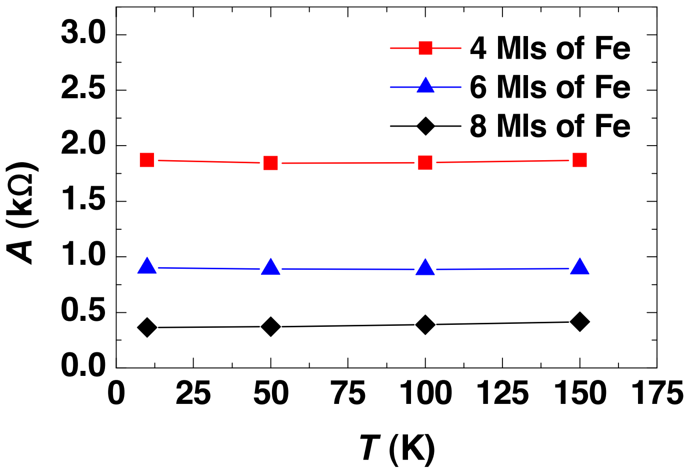

Supplementary Figure 3: **Temperature dependence of A.** Temperature dependence of the isotropic part of the longitudinal resistivity (parameter A) for different numbers of Fe monolayers.

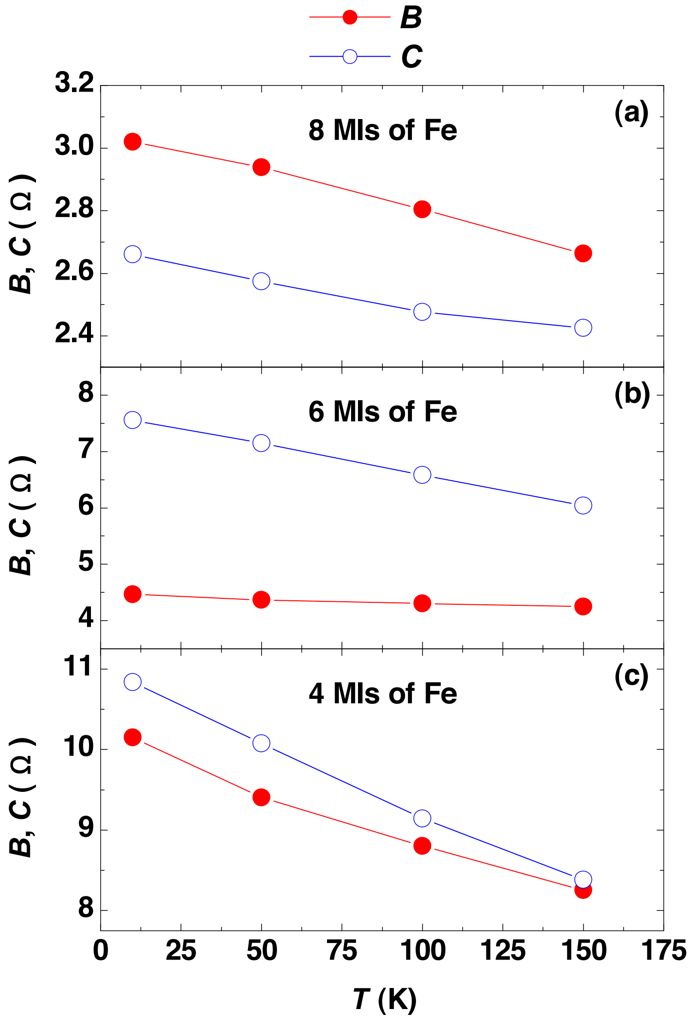

Supplementary Figure 4: **Temperature dependence of B and C.** Temperature dependence of the parameters B and C for different numbers of Fe monolayers.

Supplementary Tables

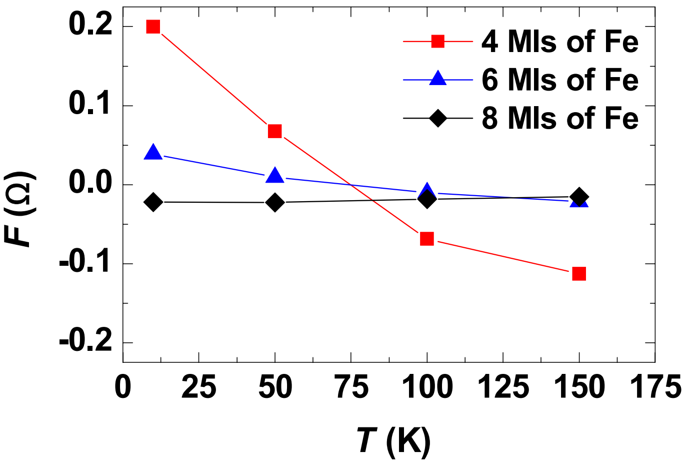

Supplementary Figure 5: **Temperature dependence of *F*.** Temperature dependence of the fitting parameter F for different numbers of Fe monolayers.

anisotropy	3 layers	6 layers	9 layers
χ_{A}	27%	25%	15%
χ_{MA}	21%	12%	5%

Supplementary Table I: Magnetoanisotropy in Fe/GaAs from first principles. Calculated anisotropy χ_{A} of the electron charge density and magnetoanisotropy χ_{MA} (both are defined by Supplementary Equation 3), as measures of the interfacial C_{2v} symmetry effects, for different numbers of Fe monolayers.

Supplementary Note 1. First principles calculations

To characterize interface induced anisotropy and magnetoanisotropy, we look closely at the electronic density $\rho(z,m) = \int \rho(x,y,z,m)\mathrm{d}x\mathrm{d}y$. For the charge anisotropy we defined the following quantities in the manuscript: the anisotropy of the charge density

$$\rho_{\mathrm{A}}(z) = \int |\rho(x,y,z,m) - \rho(y,x,z,m)|\mathrm{d}x\mathrm{d}y, \tag{1}$$

and the magnetoanisotropy of the charge density

$$\rho_{\mathrm{MA}}(z) = \int |\rho(x,y,z,m=[1\bar{1}0]) - \rho(x,y,z,m=[110])|\mathrm{d}x\mathrm{d}y. \tag{2}$$

We note that the direction x is along $[1\bar{1}0]$ and y along $[110]$, which are the principal directions of the interface induced C_{2v} symmetry (see also below).

In Supplementary Figure 1 we show $\rho(z)$ and $\rho_A(z)$ for magnetization m pointing along the [110] direction. Only the states at the Fermi level (in a window of 10 meV) are considered. We also show the magnetoanisotropy $\rho_{\mathrm{MA}}(z)$. Both ρ_{A} and ρ_{MA} decrease with increasing number of Fe monolayers, as the overlap of the Fermi level wave functions with the interface plays less role in the thicker samples. Also, the two anisotropies, ρ_{A} and ρ_{MA}, are very similar. While ρ_{A} is due to the anisotropic interfacial structure and does not depend on spin-orbit coupling, ρ_{MA} is due to the interfacial spin-orbit coupling (which arises due to the interfacial structure). Supplementary Figure 2 plots the cross section of the charge density anisotropy for the plane of [110] and [001] directions. The anisotropy in this case is given by $|\rho(x,0,z,m) - \rho(0,x,z,m)|$, for magnetization m along [110]. The strongest anisotropy is visible for the Fe d-orbitals at the interface.

As a rough estimate of the magnitudes of the crystalline and magntic anisotropies, we consider the following ratios

$$\chi_{\mathrm{A}} = \frac{\int dz\, \rho_{\mathrm{A}}(z)}{\int dz\, \rho(z)}, \qquad \chi_{\mathrm{MA}} = \frac{\int dz\, \rho_{\mathrm{MA}}(z)}{\int dz\, \rho(z)}, \tag{3}$$

of the asymmetries of the charge density and the total charge density, integrated over the whole unit cell. The results are in Supplementary Table I. As expected, both χ_{A} and χ_{MA} decrease with increasing the number of layers. The

calculated anisotropies are quite significant, showing that ideal interfaces could indeed generate large crystalline and magnetocrystalline anisotropies. The latter is still relatively large (5%) even for 9 Fe monolayers. These values could represent the experimental limits on what can be expected for transport anisotropies. Both interface imperfections and spin-flip scattering at the interfaces degrade the measured anisotropies, leading to much smaller values reported in the experiment in the main text, especially for the magnetoanisotropy, which is experimentally about a decade smaller than its theoretical limit. The crystalline anisotropy is about 5 times weaker.

Supplementary Note 2. Symmetry analysis

The conductivity tensor g_{ij} of Fe on GaAs depends explicitly on two vectors: Fe magnetization m_i and the interfacial spin-orbit field (SOF) $w_i(\mathbf{k})$ which depends on the electron momentum $\mathbf{k}$. Since the magnetoanisotropic effects (which appear due to spin-orbit coupling) are weak, we can treat the spin-orbit coupling as a perturbation and expand

$$\begin{aligned} g_{ij} &= a_{ij} + a_{kij}m_k + a_{klij}m_k m_l + b_{klij}w_k w_l \\ &+ b_{klmij}m_k w_l w_m + b_{klmnij}m_k m_l w_m w_n. \end{aligned} \tag{4}$$

Cyclotron effects are not considered; they are absent in the in-plane magnetization geometry we consider in the manuscript. Terms containing odd powers of $w_i(\mathbf{k})$ have been omitted, since they vanish upon momentum average. The expansion coefficients above are defined for an unperturbed system (in our case Fe) with $\mathrm{O_h}$ symmetry. It follows [1] that the coefficients obey the symmetry relations,

$$\begin{aligned} a_{ij} &= \tilde{S}_{io}\tilde{S}_{jp}a_{op}, \\ a_{kij} &= \det(S)S_{kq}S_{io}S_{jp}a_{qop}, \\ a_{klij} &= \det(S)^2 S_{kq}S_{lr}S_{io}S_{jp}a_{qrop}, \\ b_{klij} &= \det(S)^2 S_{kq}S_{lr}S_{io}S_{jp}b_{qrop}, \\ b_{klmij} &= \det(S)^3 S_{kq}S_{lr}S_{ms}S_{io}S_{jp}b_{qrsop}, \\ b_{klmnij} &= \det(S)^4 S_{kq}S_{lr}S_{ms}S_{nt}S_{io}S_{jp}b_{qrstop}, \end{aligned} \tag{5}$$

where the matrices S with elements S_{ij} refer to the generators of the cubic symmetry $\mathrm{O_h}$ [1]. In order to account for the orbital effects (i.e. independent of spin-orbit coupling) resulting from the two-fold anisotropic interfacial structure we have further imposed the invariance of a_{ij} under $\mathrm{C_{2v}}$ symmetry operations, whose generators are represented by the matrices $\tilde{S}$ with elements $\tilde{S}_{ij}$.

The conductivity must also obey the Onsager relation $g_{ij}(\mathbf{m}) = g_{ji}(-\mathbf{m})$, which imposes further constraints on the expansion coefficients. Solving the system of equations generated by the constraints on the expansion coefficients we find, after averaging (denoted as $\langle ... \rangle$) over the Fermi momenta, the general form of the conductivity. Inverting the conductivity and taking into account that the anisotropic contributions are small, the resistivity tensor for lateral transport in (x, y) plane has the form,

$$\rho = (r_0 - Q_+)\,\mathrm{I} - r_2 m_x m_y \sigma_x - (Q_- + r_5)\,\sigma_z, \tag{6}$$

where

$$Q_\pm = r_3^\pm + \left(r_1 + r_4^+\right)\frac{\left(m_x^2 \pm m_y^2\right)}{2} + r_4^- \frac{\left(m_x^2 \mp m_y^2\right)}{2}. \tag{7}$$

In Supplementary Equation. (6), I represents the (2×2) unit matrix and σ_x, σ_y, and σ_z are the Pauli matrices. The r-coefficients entering the expressions for the resistivity are given by,

$$r_i = \langle c_i \rangle r_0^2 \ , \tag{8}$$

and

$$r_i^\pm = \langle c_i (w_x^2 \pm w_y^2)/2 \rangle r_0^2 \ , \tag{9}$$

where

$$c_0 = \frac{(a_{11} + a_{22})}{2} + a_{1122} + (b_{1122} + b_{111122})|\mathbf{w}|^2 \ , \tag{10}$$

$$c_1 = a_{1111} - a_{1122} + (b_{112211} - b_{111122})|\mathbf{w}|^2 \;, \tag{11}$$

$$c_2 = 2a_{1212} + 2b_{121112}|\mathbf{w}|^2 \;, \tag{12}$$

$$c_3 = b_{1111} - b_{1122} + b_{112222} - b_{111122}; \;, \tag{13}$$

$$c_4 = b_{111111} + b_{111122} - (b_{112211} + b_{112222}) \;, \tag{14}$$

and

$$c_5 = \frac{(a_{11} - a_{22})}{2}. \tag{15}$$

The relevant physical quantity is the longitudinal resistivity,

$$\rho_{\text{long}} = \frac{E_{\text{long}}}{J} = \rho_{11} j_x^2 + \rho_{22} j_y^2 + (\rho_{12} + \rho_{21}) j_x j_y, \tag{16}$$

which relates the electric field along the current direction, $E_{\text{long}} = (\hat{\mathbf{j}} \cdot \mathbf{E})$, and the amplitude J of the in-plane current density $\mathbf{J} = J\hat{\mathbf{j}}$. In our case subscripts x (or 1) and y (or 2) denote the $[1\bar{1}0]$ and $[110]$ directions, respectively.

Using the resistivity tensor given by Supplementary Equation (6) we obtain the longitudinal resistance, for a sample of length L along, and width W transverse to the current path,

$$R(\theta,\phi) = \frac{L}{W}\rho_{\text{long}}(\theta,\phi) \approx A + B\cos^2(\phi-\theta) - \frac{B-C}{2}\cos(2\theta)\cos(2\phi) - (D+F+G)\cos(2\theta) - F\cos(2\phi), \tag{17}$$

where

$$A = \left(r_0 - r_3^+\right)\frac{L}{W} - \frac{B-C}{2} \;, \;\; B = -r_2\left(\frac{L}{W}\right) \;, \;\; C = -\left(r_1 + r_4^+\right)\frac{L}{W} \;, \tag{18}$$

and

$$D = r_3^-\left(\frac{L}{W}\right) \;, \;\; F = \frac{r_4^-}{2}\left(\frac{L}{W}\right) \;, \;\; G = r_5\left(\frac{L}{W}\right). \tag{19}$$

The parameters D and F carry information about the anisotropy induced by the interface SOF. Distinctly, G accounts for the intrinsic anisotropy of the interfacial structure and does not depend on spin-orbit coupling. In the absence of the interface (i.e., in the limits $\mathbf{w} \to 0$ and $a_{11} \to a_{22}$) D, F, and G vanish and the longitudinal resistance [see Supplementary Equation (17)] reduces to that of the bulk Fe.

For C_{4v} (pure Bychkov-Rashba) and D_{2d} (pure Dresselhaus)-symmetric SOFs the parameters r_i^- vanish, as can be explicitly shown by substituting $w_x = (\beta - \alpha)k_y$ and $w_y = (\beta + \alpha)k_x$ for the SOF. However, if the symmetry of the SOFs is reduced to that of the C_{2v} point group, the parameters r_i^- are, in general, finite. In particular, when the C_{2v} symmetry of the SOF is due to the interference between Bychkov-Rashba and Dresselhaus terms, one obtains

$$r_i^- \propto \alpha\beta. \tag{20}$$

Therefore, the C_{2v} symmetry of the interface SOF leads to finite values of the parameters D and F. The finite value of F gives rise to the two-fold symmetry of the CAMR of Fe on GaAs reported in the manuscript. The remaining parameters, A, B, and C do not depend on the r_i^--coefficients. Therefore they do not contain information about the two-fold symmetry of the interface SOFs.

As introduced in the manuscript, our CAMR is given by,

$$\text{CAMR}(\theta) \approx \frac{B + C + (C-B)\cos(4\theta) - 4F\cos(2\theta)}{4A}. \tag{21}$$

The reorientation of the symmetry axes of the CAMR, reported in the main text as the result of decreasing the number of Fe monolayers, can be qualitatively explained by using our phenomenological model. According to Supplementary

Equation (21) the orientation of the symmetry axes of the CAMR is determined by the sign of the difference $C - B$, which in terms of the SOF and the expansion coefficients reads as,

$$\begin{aligned} C - B &= \langle 2a_{1212} + a_{1122} - a_{1111} \rangle A^2 \frac{L}{W} \\ &- \langle (b_{111111} + b_{112211} - b_{111122} - b_{112222} - 4b_{121112}) \, |\mathbf{w}|^2 \rangle \frac{A^2}{2} \frac{L}{W}. \end{aligned} \quad (22)$$

If the Fe layer is thick enough the wave function penetration into the Fe/GaAs interface is small and the average interface SOF is negligible. In such a case, the sign of $C - B$ is determined by the first contribution on the right-hand-side of Supplemetary Equation (22). If the Fe layer becomes thinner, the average interface SOF starts to be relevant and the two contributions to $C - B$ compete. For a thin enough Fe layer the SOF-dependent contribution can eventually dominate and invert the sign of $C - B$, causing the reorientation of the symmetry axes of the CAMR.

Supplementary Note 3. Phenomenological parameters: fits to experimental data

Using Supplementary Equations (17) and (21), and the experimental values of CAMR at $\theta = 0, \pi/4, \pi/2$ together with the measured resistance at $\theta = \phi = \pi/4$, one can extract the values of our phenomenological parameters by solving the following system of linear equations,

$$\begin{aligned} A + B &= R(\pi/4, \pi/4) \\ \frac{C - 2F}{2A} &= \text{CAMR}(0) \\ \frac{B}{2A} &= \text{CAMR}(\pi/4) \\ \frac{C + 2F}{2A} &= \text{CAMR}(\pi/2). \end{aligned} \quad (23)$$

In the following we discuss these phenomenological parameters, as well as their extracted temperature dependence. Supplementary Figure 3 shows the isotropic part of the resistance (parameter A). The isotropic contribution increases when decreasing the number of Fe monolayers; it does not depend appreciably on temperature.

In Supplementary Figure 4 we show the temperature dependence of the parameters B and C. They are three orders of magnitude below the isotropic parameter A. Both B and C decrease when increasing temperature. A similar behavior is accordingly seen in the CAMR. Since A is quite insensitive to the temperature changes, the temperature dependence of the CAMR is dominated by the sum $B + C$ [see Supplementary Equation (21)].

Both B and C increase when decreasing the number of Fe layers, but at different rates. For example, for the case of 8 Fe monolayers $B > C$ but C becomes larger than B for samples with 6 and 4 monolayers of Fe. As discussed above and in the manuscript, this behavior results in the 45°-rotation of the symmetry axes of the CAMR when decreasing the number of Fe monolayers from 8 to 6 and 4 [see Fig. 4 of the manuscript].

The parameter F, which characterizes the C_{2v} symmetry of the CAMR is displayed in Supplementary Figure 5 as a function of temperature. For the thinnest sample, this parameter is comparable to the difference $B - C$ which characterizes the fourfold symmetry. For thicker samples F is much weaker, quantifying the weakening of the interface effects. In the 8 and 6 Fe monolayers samples the increasing of the temperature leads to the vanishing of F and therefore to the suppression of the two-fold symmetry of the CAMR. However, for the case of 4 Fe monolayers the effects of the interface SOC after thermal average appear to be more robust and the two-fold symmetry of the CAMR remains appreciable even at 150 K. Interestingly, in the 4 Fe monolayers case the parameter F can change its sign when the temperature is varied. The sign change of F causes a 90°-rotation of the symmetry axes of the CAMR. This is clearly seen in Fig. 4(c) of the manuscript, in which the maximum of the CAMR occurs along the [110] direction for 10 K and 50 K (in this region $F > 0$) but along the [$1\bar{1}0$] direction for 100 K and 150 K (in this region $F < 0$). This is remarkable because it indicates that not only the structural properties but also the temperature can have an impact on the symmetry properties of magnetoanisotropic phenomena such as the CAMR.

Supplementary References

---

[1] Birss, R. R. Symmetry and magnetism. *North-Holland, Amsterdam* (1966).
